# Transitioning to a Personalized Approach in Molecularly Subtyped Small-Cell Lung Cancer (SCLC)

**DOI:** 10.3390/ijms25084208

**Published:** 2024-04-10

**Authors:** Anna Grenda, Paweł Krawczyk, Adrian Obara, Łukasz Gajek, Aleksandra Łomża-Łaba, Janusz Milanowski

**Affiliations:** 1 Department of Pneumonology, Oncology and Allergology, Medical University of Lublin, 20-950 Lublin, Poland; pawel.krawczyk@umlub.pl (P.K.); aleksandra.lomza98@gmail.com (A.Ł.-Ł.); janusz.milanowski@umlub.pl (J.M.); 2 Institute of Genetics and Immunology Genim LCC, Filaretów 27/2, 20-609 Lublin, Poland; obara12lb@interia.pl (A.O.); luk.gajek@gmail.com (Ł.G.)

**Keywords:** small-cell lung cancer, molecular types, transcriptome pattern, immunotherapy, molecularly targeted therapies

## Abstract

Lung cancer has become a major public health concern, standing as the leading cause of cancer-related deaths worldwide. Among its subtypes, small-cell lung cancer (SCLC) is characterized by aggressive and rapid growth, poor differentiation, and neuroendocrine features. Typically, SCLC is diagnosed at an advanced stage (extensive disease, ED-SCLC), with distant metastases, and is strongly associated with tobacco smoking and has a poor prognosis. Recent clinical trials, such as CASPIAN and IMpower133, have demonstrated promising outcomes with the incorporation of immune checkpoint inhibitors in first-line chemotherapy, leading to prolonged progression-free survival and overall survival in patients with ED-SCLC compared to standard chemotherapy. Other studies have emphasized the potential for future development of molecularly targeted therapies in SCLC patients, including inhibitors of IGF-1R, DLL3, BCL-2, MYC, or PARP. The molecular subdivision of SCLC based on transcriptomic and immunohistochemical analyses represents a significant advancement in both diagnostic and clinical approaches in SCLC patients. Specific molecular pathways are activated within distinct transcriptome subtypes of SCLC, offering the potential for personalized treatment strategies, such as targeted therapies and immunotherapies. Such tailored approaches hold promise for significantly improving outcomes in SCLC patients.

## 1. Introduction

Small-cell lung cancer (SCLC) accounts for approximately 15% of all lung cancer cases. It is distinguished by an extremely rapid growth rate, high susceptibility to early metastasis, and poor prognosis. The primary etiological factor for SCLC is exposure to carcinogens present in cigarette smoke [1]. Patients diagnosed with small-cell lung cancer often have massive enlargement of the mediastinal lymph nodes, leading to symptoms such as coughing and dyspnea. In addition, they frequently experience manifestations associated with distant metastases, such as weight loss, weakness, bone pain, and neurological disorders [2]. In histological analysis, SCLC cells are round, oval, or spindle-shaped with a small amount of cytoplasm. These cells demonstrate a high frequency of mitotic division and typically grow in clusters, lacking the architectural organization characteristic of adenocarcinoma or squamous cell carcinoma [3].

Progress in the treatment of small-cell lung cancer has been notably slower compared to non-small-cell lung cancer (NSCLC). The standard treatment regimen for limited-stage SCLC has remained largely unchanged for over two decades, typically involving four to six cycles of chemotherapy with cisplatin and etoposide administered concurrently with thoracic radiotherapy, followed in most cases by prophylactic cranial irradiation (PCI). Median survival rates range from approximately 25 to 30 months, with one-third of patients surviving five years after diagnosis [4]. The prognosis in patients with extensive-stage SCLC has historically been significantly poorer, with survival rarely exceeding 12 months from diagnosis. After many years of unsuccessful attempts to improve outcomes for patients with extensive-stage small-cell lung cancer, an innovative approach has emerged—the use of immunotherapy in combination with chemotherapy, known as chemoimmunotherapy. This treatment strategy has prolonged overall survival (OS) and progression free survival (PFS) compared to traditional chemotherapy alone. Currently, in many European Union countries, the standard first-line treatment for patients with extensive-stage SCLC (ED-SCLC) involves the use of antibodies targeting programmed death ligand 1 (PD-L1)—atezolizumab or durvalumab—in combination with chemotherapy based on platinum compounds and etoposide [5]. Modern personalized therapeutic strategies that would align with advancements in diagnostics based on sophisticated molecular biology techniques like transcriptomics are sorely needed in small-cell lung cancer management. These methodologies have been used in clinical trials analyzing the efficacy of immunotherapy in SCLC cohorts. In our article, we address molecular–clinical issues that may lead to the implementation of SCLC molecular subtype classification in routine clinical practice, enabling personalized treatment of SCLC patients. At present, there is no predictive factor to qualify SCLC patients for targeted therapies or immunotherapy. Unlike in NSCLC, where PD-L1 (programmed cell death ligand 1) serves as a reliable marker of the efficacy of immune checkpoint inhibitors, no correlation between the expression of this molecule on tumor cells and the efficacy of immunotherapy in small-cell lung cancer has been established. Therefore, it is important to distinguish between the molecular subtypes of this molecularly complex and clinically aggressive cancer as this could enable a more personalized approach tailored to the specific molecular characteristics of each subtype.

## 2. Immunochemotherapy in SCLC Patients

A randomized, controlled, and open-label phase 3 trial (CASPIAN) evaluated the efficacy of durvalumab in combination with chemotherapy versus chemotherapy alone as the first-line treatment of ED-SCLC. A total of 268 patients were included in the chemoimmunotherapy arm, and 269 in the chemotherapy arm. The primary endpoint of the study was overall survival. The results showed that, in the durvalumab plus chemotherapy group, the median OS was longer (13.0 months) compared to the chemotherapy group (10.3 months), with a hazard ratio (HR) of 0.73 (95% CI: 0.59–0.91) [6].

In the phase 3 KEYNOTE-604 clinical trial, 228 ED-SCLC chemotherapy-naïve patients were randomized to the pembrolizumab plus chemotherapy group and 225 patients to the chemotherapy group. Pembrolizumab in combination with chemotherapy significantly improved PFS (HR = 0.75; 95% CI: 0.61–0.91) compared to chemotherapy alone. However, the median OS was similar in both groups of patients (10.8 months in the chemoimmunotherapy group and 9.7 months in the chemotherapy group) [7].

The IMpower133 trial was a double-blind study that evaluated the efficacy and safety of atezolizumab in combination with carboplatin and etoposide in comparison to carboplatin and etoposide as the first-line treatment of ED-SCLC patients [8]. A total of 201 patients were included in the chemoimmunotherapy group and 202 patients in the chemotherapy group. Median PFS was longer in the chemoimmunotherapy group (5.2 months) compared to the chemotherapy group (4.3 months), with a hazard ratio for disease progression of 0.77 (95% CI: 0.62–0.96). Moreover, the time to intracranial progression was nearly twice as long in the chemoimmunotherapy group compared to the chemotherapy group. The median OS was 12.3 months in the chemoimmunotherapy group, which was significantly higher than in the chemotherapy group (10.3 months, HR = 0.76, 95% CI: 0.60–0.95) [8,9]. Adverse events were reported in 188 patients (94.9%) in the chemoimmunotherapy group and 181 patients (92.3%) in the placebo group. The most common grade 3 or 4 treatment-related adverse events were neutropenia, anemia, and decreased neutrophil count [8].

IMbrella, an open-label, multicenter, randomized, extension, and long-term observational study, allowed further follow up of overall survival in patients treated with atezolizumab in the IMpower133 study [10]. The patients were eligible for inclusion in the rollover study if they either continued atezolizumab treatment at the end of the IMpower133 study or had already completed atezolizumab treatment and remained under observation. Among the 201 patients from the IMpower133 and IMbrella studies, one-year survival was observed in 52% of the subjects and 5-year survival in 12% of the subjects [11]. In comparison, in the group of patients who received chemotherapy alone in the IMpower133 study, one-year survival was observed in 39% of the subjects. Unfortunately, information regarding 5-year survival in this group of patients was not available. The differences in the median of PFS and OS in SCLC patients treated with standard methods and with the use of personalized treatment methods appear to be small. This is due to the lack of predictive factors in qualification of SCLC patients for immunotherapy or molecularly targeted therapies. A small percentage of patients benefit from adding immunotherapy to chemotherapy for several years. The IMbrella study was designed to investigate the molecular profile of SCLC in these patients. Unfortunately, the size of the population qualified for this study was too small and this clinical trial ended inconclusively.

## 3. Transcriptional Profile of SCLC and Its Relationship with the Effectiveness of Immunotherapy

In the IMpower133 trial, the primary endpoints were PFS and OS, but the study also provided valuable molecular information on the expression pattern of SCLC-related genes. Transcriptional subtypes of SCLC were determined based on next-generation sequencing (NGS) at the RNA level, identifying distinct categories such as SCLC-A (ASCL1-driven), SCLC-N (NEUROD1-driven), SCLC-P (POU2F3-driven), and SCLC-I (inflamed). The frequency of transcriptional subtypes based on RNA sequencing in the available patient cohort (*n* = 271) in the IMpower133 study was as follows: SCLC-A—51.7%, SCLC-N—22.5%, SCLC-P—7.7%, and SCLC-I—18.1% [12].

The distribution of transcriptional subtypes based on RNA sequencing from the IMpower133 trial was available for seven of the eleven subjects from the IMbrella trial who survived beyond 5 years. Among these patients, one had the SCLC-A subtype (14.3%), four had the SCLC-N subtype (57.1%), and two had the SCLC-I subtype (28.6%). None of the patients showed the SCLC-P subtype. In the entire IMpower133 cohort, the patients with the SCLC-I type exhibited the longest median OS in the chemoimmunotherapy group compared to the chemotherapy group (18.2 vs. 10.4 months, respectively), indicating the lowest risk of death compared to patients with other transcriptional subtypes [12].

An interesting observation concerning the *TP53* (tumor protein P53) and *RB1* (RB transcriptional corepressor 1) signatures was made by George et al. (2015) in their whole-genome sequencing study. The authors observed a high mutation rate in SCLC and a very high prevalence of pathological variants in the *TP53* and *RB1* genes [13]. Gay et al. (2021) indicated that *MYC* (*MYC proto-oncogene*, *BHLH transcription factor*) amplifications could be preferentially associated with the SCLC-P subtype as its expression was significantly higher in patients with this subtype [12].

Transcriptional subtypes are determined based on the expression patterns of genes, and it is important to consider their cellular functions as they may directly impact the pathogenesis and progression of SCLC. Achaete-scute homolog 1, a transcription factor encoded by the *ASCL1* gene, is involved in neuronal differentiation, mainly of autonomic and olfactory nerve cells. Overexpression of ASCL1 has been implicated in the development of medullary thyroid cancer and small-cell lung cancer [14]. According to Lee et al. (2020), neurogenic differentiation 1, a transcription factor encoded by the *NEUROD1* gene, is responsible for the transformation of reactive glial cells into functional neurons and regulates the expression of the insulin-encoding gene. Mutations in *NEUROD1* have been associated with the development of type II diabetes [15]. POU domain class 2 transcription factor 3, encoded by the *POU2F3* gene, plays a role in keratinocyte differentiation and proliferation. A lack of *POU2F3* expression due to methylation and subsequent silencing may be responsible for the formation of cervical cancer [16].

Recent studies have classified SCLC into high- and low-neuroendocrine (NE) subtypes based on their ability to produce neuroendocrine factors and the relative expression of transcription factors. Neuroendocrine SCLC cells typically express markers such as chromogranin A, synaptophysin, and CD56 antigen, which are routinely assessed in the immunohistochemical diagnostics of small-cell lung cancer. However, utilizing transcription factor expression to determine SCLC represents a significant and groundbreaking classification method that may serve as a foundation for personalized diagnosis and treatment of SCLC patients. Nevertheless, many questions remain regarding the validation of the results that gave rise to this classification. Lissa et al. (2022) pointed out the limited availability of high-quality tumor tissue materials for genomic and transcriptomic analysis [17]. They also indicated that certain types of materials (e.g., thin-needle aspirates) may not provide adequate quantity and quality of cells and thus RNA for high-throughput transcriptomic analyses. Moreover, rapid tumor progression and occurrence of multiple metastases and comorbidities may hinder the acquisition of biopsy materials. Lissa et al. (2022), in their evaluation of metastatic sites, histology, transcriptome, and exome profile, as well as treatment outcomes of SCLC patients, demonstrated complex intra- and intertumoral heterogeneity of neuroendocrine differentiation. Transcriptomic analysis confirmed the subtypes previously described by Rudin et al., which were based on ASCL1, NEUROD1, POU2F3, and YAP1 expression. The YAP1 subtype corresponds to the SCLC-I subtype, previously described by Gay et al. (2021). Subtypes with mixed NE and non-NE phenotypes are characterized by chemotherapy resistance and inferior outcomes [17]. Lissa et al. (2022) estimated the frequency of SCLC subtypes as follows: SCNC-A subtype in thirty patients (41.7%), SCNC-Y subtype in twenty-one patients (29.2%), SCNC-N subtype in twenty patients (27.8%), and SCNC-P subtype in one patient (1.4%). The authors showed that the expression of the transcription factor YAP1 (Yes1-associated transcriptional regulator) was higher in tumors with non-NE differentiation. On the other hand, neuroendocrine tumors were described as having a higher likelihood of harboring mutations in *RB1*, *NOTCH* (Notch receptor 1), and chromatin-modifying genes, along with upregulation of DNA damage response genes. These tumors showed greater sensitivity to therapies targeting replication stress [17]. Furthermore, patients with non-NE tumors were more likely to respond to immunotherapy [17].

Certain SCLC subtypes do not exhibit neuroendocrine differentiation at the transcriptional level, and they belong to the SCNC-Y and SCNC-P subtypes, in which chromogranin, synaptophysin, and INSM1 (INSM transcriptional repressor 1) immunochemical tests may be negative. In contrast, increased activity of cellular pathways related to immune function, cell adhesion, or metabolism is observed in these subtypes, with genes displaying the increased molecular activity listed in Table 1. Kim et al. (2023) identified several markers overexpressed in the heterogenous non-NE SCLC subtype, including *IFITM3* (interferon-induced transmembrane protein 3), *B2M* (beta-2-microglobulin), *ANXA4* (annexin A4), *VIM* (vimentin), *CD74*, *S100A11* (S100 calcium binding protein A11), and *YAP1* [18]. Qu et al. (2022) also reported tumor heterogeneity, with ASCL1, NEUROD1, POU2F3, and YAP1 being the predominant molecular SCLC subtypes with frequencies of 78.2%, 5.6%, 7%, and 2.8%, respectively. However, 6.3% of the tumors were negative for the expression of all four transcription factors. Tumors other than SCLC-A/N were characterized by greater infiltration of CD8+ T cells and more often displayed an “inflammatory” immunophenotype. Nonetheless, the researchers emphasized that their classification was based on IHC in primary SCLC tumors, suggesting that other subtypes may be characteristic of metastatic tumors as tumor molecular subtypes may continue to evolve [19].

There are indications that immunotherapy in patients with the Y and P subtypes may be more effective compared to those with the other two SCLC subtypes as they can be more sensitive to DNA-damaging drugs such as platinum compounds. Nevertheless, certain genes associated with response to immunotherapy, such as *B2M* and *TGFB1*, when highly expressed, have been associated with failure of immune checkpoint inhibitors (*B2M* and *TGFB1*). It is possible that specific components of SCLC, although in the minority, showing overexpression of these genes, may develop resistance to immune checkpoint inhibitors (ICI). Generally, from a molecular perspective, fewer transcriptionally neuroendocrine cells improve the chances of successful ICI treatment in SCLC patients. The molecular subtype shows predominant expression of a particular transcription factor, with heterogeneity within the tumor partly attributable to the plasticity and gradual evolution of tumor cells [32]. However, the most crucial aspect is the advancement of targeted therapy development for SCLC patients. These therapies aim to target specific molecular pathways activated in particular transcriptome subtypes of SCLC. This marks a significant shift after a prolonged period often referred to as the “molecular treatment desert” in SCLC.

## 4. Potential Targeted Treatment in Molecular SCLC Subtypes

In the SCLC-A subtype, potential targets include BCL-2 (B cell lymphoma 2) and delta-like protein 3 (DLL3). ASCL1 expression is correlated with high DLL3 and BCL-2 expression. The first one decreases the expression of NOTCH receptors, which is associated with the loss of suppressor functions of these proteins. Therefore, potential therapeutic options for SCLC-A patients include BCL-2 inhibitors or antibodies against DLL3. Rovalpituzumab tesirine (Rova-T) is an antibody–drug conjugate containing a DLL3-targeting antibody tethered to pyrrolobenzodiazepine, an agent cytotoxic to tumor cells. Unfortunately, the TAHOE phase 3 (Rova-T vs. topotecan for second-line treatment in SCLC patients with high DLL3 expression) and MERU phase 3 (Rova-T compared to placebo for maintenance therapy) clinical trials did not demonstrate the efficacy of this drug [33,34]. Some SCLC patients treated with BCL-2 inhibitors (palcitoclax, obatoclax, and navitoclax) have shown partial responses or disease stabilization, although the effectiveness of these drugs has been demonstrated in early-phase clinical trials [35]. Uprety et al. (2021) emphasized the need to distinguish SCLC transcriptome subtypes for precise patient qualification from molecularly targeted treatments. For example, preliminary evidence suggests that Aurora kinase inhibitors, BCL-2 inhibitors, PARP inhibitors, and ICI may exert differential effects on individual SCLC subtypes [36]. This aligns with the emerging potential application of targeted therapies in SCLC patients.

*MYC* amplification has been previously linked to the SCLC-P subtype. Wang et al. (2022) identified *MYC* as highly transcriptionally active in the NEUROD1 subtype of SCLC, indicating potential utilization of molecular targeting of downstream proteins. Aurora kinases, mTOR (mammalian target of rapamycin), and IMDPH (inosine monophosphate dehydrogenase 1) inhibitors may have therapeutic potential in these SCLC types [32].

Li et al. (2023) reported that certain SCLC subtypes were highly sensitive to Aurora kinase inhibitors, yet early-phase studies showed a short-lasting response, suggesting that effective therapeutic combinations need to be identified to prolong their efficacy [37]. The authors used a mouse xenograft model to test the efficacy of combining Aurora kinase inhibitor and anti-PD-L1 immunotherapy. Their findings demonstrated sustained efficacy of the highly specific Aurora kinase inhibitor A (LSN3321213) and anti-PD-L1 antibody. LSN3321213 induced accumulation of tumor cells in the mitotic phase, characterized by low *ASCL1* expression and high expression of genes associated with the interferon pathway and antigen presentation, mimicking the inflammatory SCLC subtype. These data demonstrated that the expression of inflammatory genes was restored during the mitotic phase in tumor cells, suggesting the potential of inhibiting Aurora kinase A as a therapeutic strategy [37].

Kern et al. (2020) reported that mTOR inhibitors in monotherapy significantly suppressed SCLC growth and sensitized SCLC to cisplatin and etoposide chemotherapy. Moreover, they observed that mTOR inhibitors delayed or potentially averted the development of chemoresistance [38]. In addition, SCLC with *MYC* amplification was found to be sensitive to arginine deficiency and IMPDH inhibition [39,40].

In the SCLC-P subgroup, PARP (poly ADP ribose polymerase) and IGF-1R (insulin-like growth factor 1 receptor) inhibitors, along with treatment based on nucleoside analogues, seem to be potential therapeutic options. In a randomized phase 2 study, 181 ED-SCLC patients received veliparib plus platinum-based chemotherapy as the first-line treatment, followed by veliparib as a maintenance therapy. A modest improvement in PFS was demonstrated without a corresponding benefit in OS. The group receiving veliparib in combination with chemotherapy had a median PFS of 5.8 months, while the median PFS in the subjects administered placebo plus chemotherapy, followed by the placebo, reached 5.6 months [41]. Another randomized, double-blind phase 2 trial evaluated whether adding veliparib to temozolomide (TMZ) would improve 4-month disease progression-free survival in 104 patients with relapsed SCLC [42]. The study did not reveal a significant difference in the percentage of patients with 4-month PFS between the TMZ and veliparib group (36% of patients) and the TMZ and placebo group (27% of patients). Median OS also did not differ significantly between these groups. However, the objective response rate (ORR) was significantly higher in the subjects receiving TMZ and veliparib (39% of patients) compared to the subjects treated solely with TMZ (14% of patients) [42]. Patients from the TMZ and veliparib group with SLFN11 (Schlafen family member 11)-positive tumors demonstrated significantly higher median PFS (5.7 versus 3.6 months) and median OS (12.2 versus 7.5 months) compared to the subjects administered TMZ alone [42].

**Figure 1:**
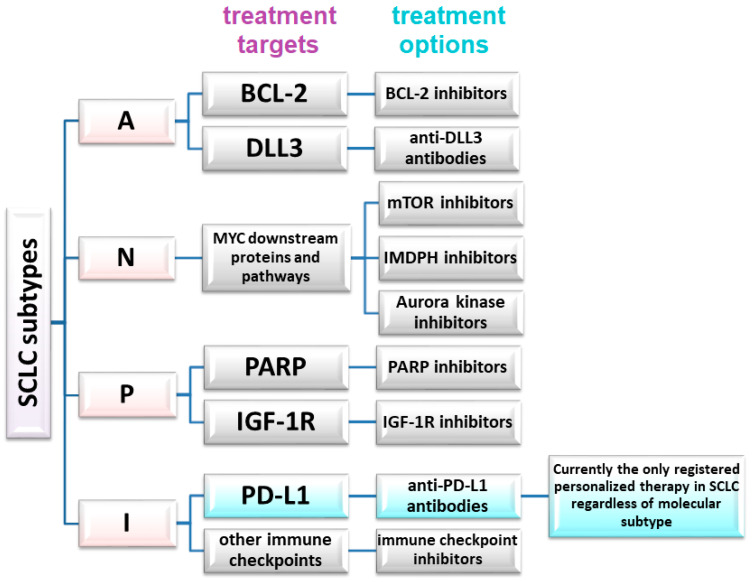
Potential therapeutic targets and treatment methods in a molecular personalized approach in SCLC.

The absence of significant differences in PFS or OS, or failures observed in trials involving, for instance, anti-DDL antibodies or other targeted therapies, also demonstrate that not all eligible SCLC patients will benefit from these therapies. However, the differences between SCLC subtypes, coupled with the capacity of SCLC to transition between subtypes in response to therapy (e.g., chemotherapy), strongly advocate for personalized therapies in small-cell lung cancer [32]. Patient selection must be based on relevant markers, such as SLFN11 expression in tumor cells. Proposed markers for the molecular classification of SCLC and potential predictive factors are shown in table in Section 6 and Figure 1.

## 5. Inflamed SCLC Subtypes—Most Suitable for Immunotherapy

ICI therapy could be the most suitable option for patients with inflamed SCLC. A study by Gay et al. (2021) identified the inflamed SCLC type (SCLC-I) in 18.1% of the patients based on the distribution of transcriptional subtypes in the full RNAseq-evaluable IMpower133 cohort (*n* = 271). In addition, SCLC-I in the IMbrella study was detected in 28.6% of the patients. While a small group of patients was included in this study, it might provide a starting point for developing a diagnostic test to distinguish molecular SCLC subtypes, with a particular focus on the inflammatory SCLC type. Further research involving larger SCLC patient cohorts treated with ICI is necessary, wherein both the transcriptome and expression of individual markers are simultaneously assessed using NGS and IHC approaches. Additionally, the presence and activation of tumor-infiltrating lymphocytes (TIL) could serve as a supportive indicator for these predictive markers.

Shirasawa et al. (2023) conducted a study investigating the association between SCLC subtypes and CD8-positive TIL status [43]. Moreover, the authors examined the association between the efficacy of atezolizumab in combination with carboplatin and etoposide therapy, SCLC subtypes, and TIL status in ED-SCLC patients. The study also included patients with localized disease (LD-SCLC). The authors identified SCLC-I as a SCLC subtype according to Gay et al.’s classification, where the inflammatory SCLC subtype (SCLC-I) was described as the fourth subtype, replacing SCLC-Y, since YAP1 expression alone did not exclusively define SCLC-I [12,43]. Transcriptomic groups were identified in 48 LD-SCLC patients, with SCLC-A + SCLC-N observed in 35% of the patients, SCLC-P in 31% of the patients, SCLC-Y in 21% of the patients, and SCLC-I in 13% of the patients [43]. The latter study revealed that SCLC-I was a subtype with high T-cell-inflamed gene expression, high CD8A (CD8 subunit alfa) expression on T lymphocytes, and activation of epithelial–mesenchymal transition (EMT). Among 85 LD-SCLC patients, the IHC method was employed to identify SCLC subtypes, and the distribution was as follows: 39% of the patients with SCLC-A, 28% with SCLC-P, 16% with SCLC-Y, and 1% with non-specific SCLC-I. The infiltration with CD8-positive TILs was the lowest in the SCLC-A + SCLC-N group and highest in the SCLC-I group [43]. Importantly, transcriptome analysis and immunohistochemistry showed some discrepancies, potentially because the former covered the expression of 1130 genes while the latter involved only four proteins [43]. The most significant deviation concerned the SCLC-Y subtype, which was also approached with caution by Gay et al. (2021). In a cohort of 42 ED-SCLC patients receiving atezolizumab plus carboplatin and etoposide therapy, IHC analysis was performed in 34 patients. Among them, 59% were classified as the SCLC-A subtype, 23% as SCLC-N, 15% as SCLC-P, and 3% as SCLC-Y. TIL infiltration was analyzed in 37 cases, and high TIL density was defined as ≥69 cells per mm^2^ of tumor tissue. The median PFS of patients with high TIL density was significantly higher compared to subjects with low TIL density (7.3 months versus 4.0 months, respectively). However, there were no differences in TIL density between SCLC subtypes.

Shirasawa et al. (2023) indicated that high TIL density, which was characteristic of the SCLC-I subtype, could predict the efficacy of atezolizumab plus carboplatin and etoposide therapy in ED-SCLC patients. However, certain pathological subtypes were not associated with the efficacy of chemoimmunotherapy [43]. Kim et al. (2023) also pointed to the tumor microenvironment as an important aspect of SCLC classification. They categorized SCLC into four types based on TME association with resistance to platinum-based therapy: SCLC-A with ASCL1 expression, SCLC-I with PD-L1 expression, SCLC-N with NEUROD1 expression, and SCLC-P with POU2F3 expression. IHC analysis showed that ASCL1 expression was most strongly attenuated in the SCLC-A subtype, but also showed reduction in the SCLC-I and SCLC-N subtypes. POU2F3 expression specifically defined the SCLC-P subtype, while NEUROD1 staining was non-specific and ineffective for classifying SCLC-N [18].

SCLC-A was associated with high expression of SCL1 (scarecrow-like protein), DLL3, FOXA1/3 (forkhead box A1/3), and SOX2, as well as with Wnt signaling. In the SCLC-I subtype, PD-L1 and T cell receptor (TCR) signaling was activated. Moreover, SCLC-I was characterized by infiltration of CD8-positive and PD-1-positive T cells, and activation of endothelial–mesenchymal transition [18]. Additionally, both the SCLC-A and SCLC-N subtypes displayed a neuroendocrine profile involving the activation of neurotransmission processes. The mitotic cell cycle was dysregulated in both the SCLC-A and SCLC-N cell lines, while PI3K-Akt signaling was activated in the SCLC-N subtype. The expression of the *POU2F3* and *MYC* genes was upregulated in the SCLC-P subtype, whereas Notch expression and extracellular matrix (ECM) reorganization were detected in the SCLC-P subtype. Additionally, the SCLC-P subtype showed activation of genes related to epithelial–mesenchymal transition [18], which was connected with worse prognosis and platinum resistance. The authors also found that the bromodomain and extra-terminal (BET) inhibitor JQ1 could overcome platinum resistance, and proposed BET inhibitors as therapeutic candidates for patients with platinum-resistant SCLC [18].

Several studies have adopted the classification developed by Gay et al. (2021) but have not confirmed the correlation between SCLC subtypes and other clinicopathological factors or immune profiles [44]. Ding et al. (2022) collected 53 samples of resectable SCLC from the primary lung tumors and identified SCLC subgroups based on IHC analysis: SCLC-A in 39.6% of patients, SCLC-N in 28.3% of patients, SCLC-P in 17.0% of patients, and SCLC-I in 15.1% of patients. They did not find a correlation between PD-L1 expression or CD8-positive TIL density and SCLC subtypes. Only Cox multivariate analysis demonstrated that lymph node metastases, TIL density, Ki-67 expression, and SCLC-P were independent prognostic factors for resectable SCLC patients [44].

## 6. Transcriptomic Analysis as an Avenue to Personalized Diagnostics and Treatment of SCLC Patients

All transcriptomic studies concerning small-cell lung cancer consistently emphasize the need to subdivide this cancer into distinct subgroups, each characterized by a unique gene expression profile with a key “leader” gene. The expression of this gene is the primary factor differentiating SCLC subgroups. Table 2 outlines the studies pertaining to SCLC division and its potential applicability in routine clinical practice. Nevertheless, there are some differences and discrepancies between these groups, which are worth considering in treatment planning.

## 7. Discussion

Lissa et al. (2022) identified SCLC-P and SCLC-Y subjects as susceptible to immunotherapy, contrasting with SCLC-A and SCLC-N patients [17]. Meanwhile, Wang et al. (2022) reported that the SCLC-Y subgroup exhibited an inflammatory phenotype [32]. In a study by Kim et al. (2023), an analysis of SCLC subtypes revealed that the inflammatory subtype (SCLC-I) demonstrated infiltration of CD8-positive and PD-1-positive T cells and exhibited a gene expression profile associated with platinum resistance. In addition, Kim et al. (2023) employed the IHC method and detected ASCL1 expression in the SCLC-A, SCLC-I, and SCLC-N subtypes, while POU2F3 clearly defined only the SCLC-P subtype. On the other hand, NEUROD1 staining was non-specific and failed to classify all the cases of the SCLC-N subtype. Additionally, TTF1+ (thyroid transcription factor 1) displayed relatively high expression in all the SCLC subtypes, except the SCLC-P subtype. Notably, *MYC* amplification and MYC expression were higher in the SCLC-I and SCLC-P subtypes [18]. Lissa et al. (2022) and Rudin et al. (2019) focused on categorizing SCLC into neuroendocrine (NE SCLC) and non-neuroendocrine (NE SCLC) types [17,45]. They highlighted potential differences in response to treatment between these two groups of patients. The latter authors concluded that patients with the non-NE SCLC subtype could benefit from immunotherapy. Gay et al. (2021) and Shirasawa et al. (2023) proposed even more precise selection of SCLC patients who could benefit from ICI. They identified the SCLC-I subgroup, in which the IHC method excluded the expression of ASCL1, NEUROD1, POU2F3, and YAP1 proteins.

**Figure 2:**
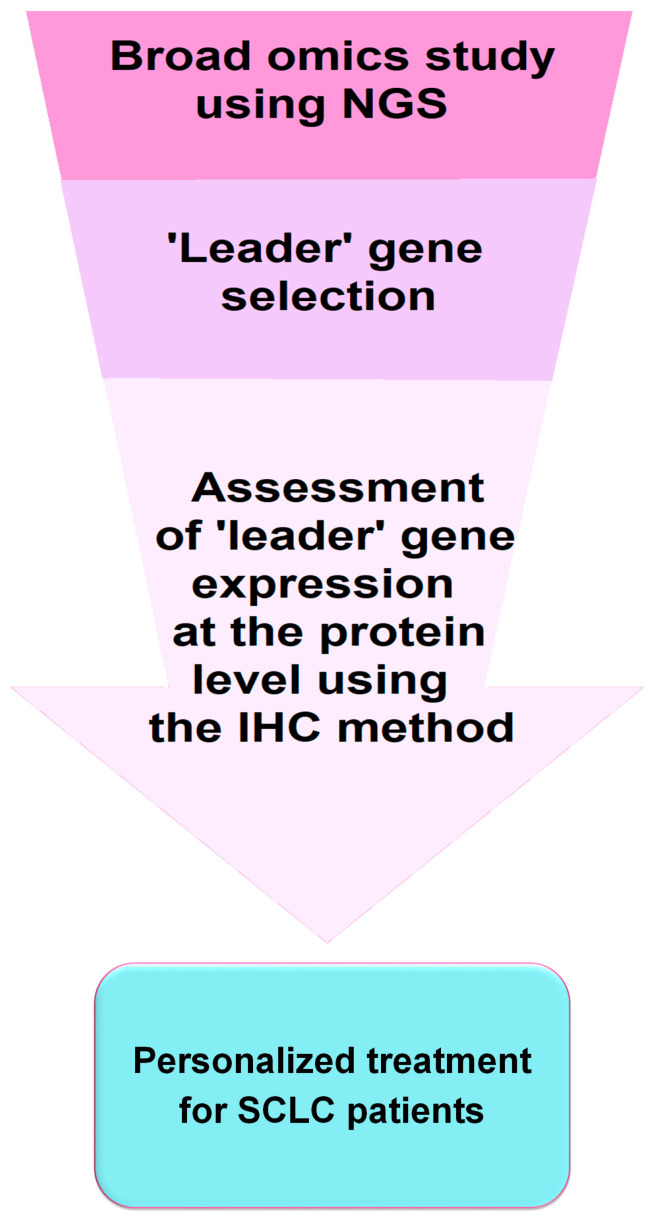
Schematic of potential implementation of a diagnostic test in personalized treatment approach for SCLC patients. It should be recognized that molecular and IHC diagnostic tests offer potential avenues for personalized treatment of SCLC, including the use of molecularly targeted therapies (e.g., BCL-2, DLL3, MYC, PARP, and IGF-1R inhibitors) and immunotherapies involving immune checkpoints inhibitors. However, particularly noteworthy are the discrepancies between transcriptomic subtypes and those identified based on IHC. Clinical trials are needed to confirm the efficacy of targeted therapies and immunotherapies in transcriptomic and pathological subtypes of SCLC. Moreover, such testing should also consider the disease stage (LD-SCLC and ED-SCLC) as the distribution of individual subtypes may change during disease progression. Nevertheless, research on SCLC is rapidly advancing, potentially paving the way for the development of personalized treatment, which could remove SCLC from the group of orphan diseases [46].

All these observations are very relevant in terms of selecting the most suitable personalized therapy for SCLC patients. Immunohistochemistry (IHC), feasible in most laboratories, seems to be appropriate for the assessment of SCLC subtypes in routine clinical practice and the qualification of patients for immunotherapy. However, genetic studies examining transcription factor expression and analysis of genetic abnormalities provide more comprehensive results and may lead to further subdivision of SCLC. Figure 2 shows a simplified scheme for implementing IHC testing into diagnostic and clinical practice for the application of personalized therapies in SCLC.

It is worth mentioning that transcriptomic analyses encompass over a thousand genetic markers, while the IHC method is significantly more limited. Thus, there is a possibility that molecular subtypes will not be perfectly translated into IHC subtypes. Further research and transcriptomic profiling are necessary to confirm that IHC correlates with the molecular profile of SCLC, enabling the implementation of immunotherapy or molecularly targeted therapies based on simple immunoassays. Qu and colleagues also highlighted other factors, such as potential differences between metastatic and non-metastatic SCLC subtypes, or the effect of prior treatments on subgroup classification results [19]. In addition, the authors have pointed out the potential necessity of multiplexed IHC due to the presence of multiple markers co-existing in tumor cells and their possible interactions. Gay et al. (2021) also suggested that subtypes could be defined and dynamically monitored using transcriptomic, proteomic, or even epigenetic approaches, offering perspectives for personalized diagnostic and clinical strategies in SCLC [12]. Artificial intelligence (AI) may be another promising approach that could help to distinguish molecular subtypes of cancers. The value of AI in lung cancer diagnosis, which is a complex and highly skilled process, is increasingly being explored in scientific studies. Liu et al. (2023) emphasized the urgent need for early lung cancer diagnosis and proposed that AI could facilitate this process through multivariate analysis [47]. AI can also prove invaluable in pathology, particularly in the analysis of small tissue samples that present significant diagnostic challenges. Shen (2021) argued that AI could be a valuable tool for introducing new molecular classification of cancers based on the molecular profile of tumors [48]. Moreover, AI can aid in histopathological diagnosis through the analysis of digital histopathologic slides and gene profiles, allowing for the differentiation of stromal cell types within the tumor microenvironment and facilitating pathological staging [49]. A comprehensive approach integrating pathomorphologic, clinical, and molecular (including transcriptomic) data can also be analyzed using AI algorithms in small-cell lung cancer. The clinical, pathological, and genetic profiles of SCLC patients could be used in patient qualification for appropriate therapy [50]. However, AI is still in development, and its use for diagnosis or qualification for personalized therapies must be preceded by the efforts of a cadre of specialized pathomorphologists, clinicians, diagnosticians, and IT specialists with extensive SCLC experience and genetic expertise.

## Figures and Tables

**Table 1:** Proteins overexpressed in the non-NE-SCLC subtype.

Protein	Function Connected with Cancers	Source
MYC (MYC proto-oncogene, BHLH transcription factor)	Multifunctional proto-oncogene involved in cell cycle promotion, apoptosis, and cellular transformation. *MYC* gene amplification is frequently observed in numerous cancers.	[20]
VIM (vimentin)	VIM is responsible for maintaining cell shape, cytoplasm integrity, and stabilizing cytoskeletal interactions. It is also responsible for cell attachment and migration. High expression is associated with metastatic cancers and poor outcomes in NSCLC patients. In patients treated with immune checkpoint inhibitors as monotherapy, survival rates are significantly higher in the vimentin-positive subjects.	[21,22]
CD8	CD8 antigen is a cell surface glycoprotein located on cytotoxic T cells. CD8-positive cytotoxic T lymphocytes are effectors in the anticancer immune response. The CD8 antigen acts as a coreceptor with the T cell receptor to recognize antigens displayed by antigen-presenting cells in the context of class I MHC molecules. Expression of this molecule is essential for the recognition of cancer cells.	[23]
HLA-B/C/E (human leukocyte antigen, class I, B/C/E)	Class I molecules play a central role in the immune response. They regulate the proliferation of tumor cells and inhibit antitumor immunity.	[24]
NOTCH	NOTCH signaling can promote or inhibit tumor development in various types of cancer. Mutations are often observed in SCLC, mainly in the *NOTCH1* gene. NOTCH may play a tumor-suppressive role in SCLC.	[25]
TGFβ1 (transforming growth factor beta 1)	TGFβ1 regulates cell proliferation, differentiation, and growth. It modulates the expression and activation of other growth factors, including interferon gamma and TNFα. It is frequently upregulated in the tumor microenvironment, causing immunosuppression. High TGFβ signaling is associated with resistance to anticancer treatments, including chemotherapies, molecularly targeted therapies, and immunotherapies.	[26]
IFITM1/2/3 (interferon-induced transmembrane protein 1/2/3)	High IFITM1 expression is associated with worse prognosis in cancer patients.	[27]
B2M (beta-2-microglobulin)	B2M, a subunit of the major histocompatibility complex (MHC) class I, plays important biological functions in tumorigenesis and immune control. Alterations in the *B2M* gene and B2M proteins have been linked to poor response to immunotherapies as they can suppress antigen presentation.	[28]
REST (RE1 silencing transcription factor)	REST acts as an oncogene or a tumor suppressor.	[29]
CD4 (T cell surface glycoprotein CD4)	CD4 is a cell surface glycoprotein expressed on T helper (Th) cells. CD4-positive T cells assist CD8-positive T cytotoxic lymphocytes in the effector phase, induce macrophages and cellular senescence of tumor cells, and disrupt tumor vasculature by releasing cytokines.	[30]
IFIT1/2/3 (interferon-induced protein with tetratricopeptide repeats 1/2/3)	IFIT1/2/3 is involved in innate immunity, antiviral immune response, virus-induced translation initiation, replication, and double-stranded RNA signaling. IFIT participates in various molecular signaling mechanisms induced by tumor progression and metastasis.	[31]

**Table 2:** Proposed SCLC subtypes.

	Proposed SCLC Subtypes and Frequency of Their Occurrence			
**SCLC Subgroups and Leading Genes**	**A (*ASCL1*)**	**N (*NEUROD1*)**	**P (*POU2F3*)**	**Y (*YAP1*)**
Rudin et al. Estimation of relative frequencies of the four subtypes based on primary tumor analysis (*n* = 81) [45]	70%	11%	16%	2%
Wang et al. Estimation of prevalence of four SCLC groups [32]	~70%	15–17%	10–15%	2–10%
Lissa et al. Transcriptomic analyses of SCLC treated with various drugs [17]	*n* = 30, 41.7%	*n* = 20, 27.8%	*n* = 1, 1.4%	*n* = 21, 29.2%
**SCLC Subgroups and Leading Genes**	**A (*ASCL1*)**	**N (*NEUROD1*)**	**P (*POU2F3*)**	**I (inflamed)**
Ding et al. IHC analysis of primary tumors from resectable SCLC patients (*n* = 53) [44]	*n* = 21, 39.6%	*n* = 15, 28.3%	*n* = 9, 17.0%	*n* = 8, 15.1%
Gay et al. ED-SCLC patients treated with atezolizumab and chemotherapy in the IMpower133 study (*n* = 271) [12]	51.7%	22.5%	7.7%	18.1%
Liu et al. ED-SCLC patients treated with atezolizumab and chemotherapy in the rollover IMbrella study [10]	*n* = 1, 14.3%	*n* = 4, 57.1%	0%	*n* = 2, 28.6%
**SCLC Subgroups and Leading Genes**	**A + N (*ASCL1* + *NEUROD1*)**	**P (*POU2F3*)**	**Y (*YAP1*)**	**I (inflamed)**
Shirasawa et al. Transcriptomic analyses in LD-SCLC patients (*n* = 48) [43]	*n* = 17, 35%	*n* = 15, 31%	*n* = 10, 21%	*n* = 6, 13%
Shirasawa et al. IHC analyses in LD-SCLC patients (*n* = 85) [43]	*n* = 44, 52% (A: *n* = 33, 39% + N: *n* = 11, 13%)	*n* = 24, 28%	*n* = 16, 19%	*n* = 1, 1% (not specified)
Shirasawa et al. IHC analyses in ED-SCLC patients (*n* = 34) [43]	*n* = 28, 82% A: *n* = 20, 59% + N: *n* = 8, 23%)	*n* = 5, 15%	*n* = 1, 3%	0%

## Data Availability

Data are contained within the article.
